# A review of current trends in three-dimensional analysis of left ventricular myocardial strain

**DOI:** 10.1186/s12947-020-00204-3

**Published:** 2020-06-26

**Authors:** Yosuke Nabeshima, Yoshihiro Seo, Masaaki Takeuchi

**Affiliations:** 1 Second Department of Internal Medicine, School of Medicine, University of Occupational and Environmental Health, 1-1 Iseigaoka, Yahatanishi, Kitakyushu, 807-8555 Japan; 2 Department of Cardiology, Graduate School of Medical Sciences, Nagoya City University, Nagoya, Japan; 3 Department of Laboratory and Transfusion Medicine, School of Medicine, Hospital of University of Occupational and Environmental Health, Kitakyushu, Japan

**Keywords:** 3D echocardiography, Speckle tracking, Left ventricular strain

## Abstract

Three-dimensional (3D) left ventricular (LV) myocardial strain measurements using transthoracic 3D echocardiography speckle tracking analysis have several advantages over two-dimensional (2D) LV strain measurements, because 3D strain values are derived from the entire LV myocardium, yielding more accurate estimates of global and regional LV function. In this review article, we summarize the current status of 3D LV myocardial strain. Specifically, we describe how 3D LV strain analysis is performed. Next, we compare characteristics of 2D and 3D strain, and we explain validation of 3D strain measurements, feasibility and measurement differences between 2D and 3D strain, reference values of 3D strain, and its applications in several clinical scenarios. In some parts of this review, we used a meta-analysis to draw reliable conclusions. We also describe the added value of 3D over 2D strain in several specific pathologies and prognoses. Finally, we discuss novel techniques using 3D strain and suggest its future directions.

## Introduction

During the last two decades, two-dimensional (2D) myocardial strain measurements from 2D echocardiography (2DE) speckle tracking analysis have become an established technique for quantifying left and right heart chamber function. Their usefulness has been validated to 1) detect subtle heart chamber dysfunction [1–3], 2) evaluate the extent and severity of heart disease [1–3], and 3) predict future outcomes in various cardiovascular pathologies [2]. All ultrasound vendors now have their own proprietary 2DE speckle tracking analytical software for the left ventricle, and some vendors have launched fully automated 2D strain analytical software employing artificial intelligence. Since the left ventricular (LV) myocardium consists of three different myocardial layers that contract in different directions simultaneously, accurate assessment requires three-dimensional (3D) analysis. Three-dimensional echocardiography (3DE) datasets with 3D speckle tracking analytical software have made it possible to perform clinical 3D strain analysis during the last decade [3–5]. 3D strain software offered by most ultrasound companies is designed to measure LV myocardial strain; thus, this review manuscript focuses only on 3D LV strain. For simplicity, strain values in each direction are presented as absolute values in the text, figures, and tables.

### 3D strain analysis

**Fig. 1:**
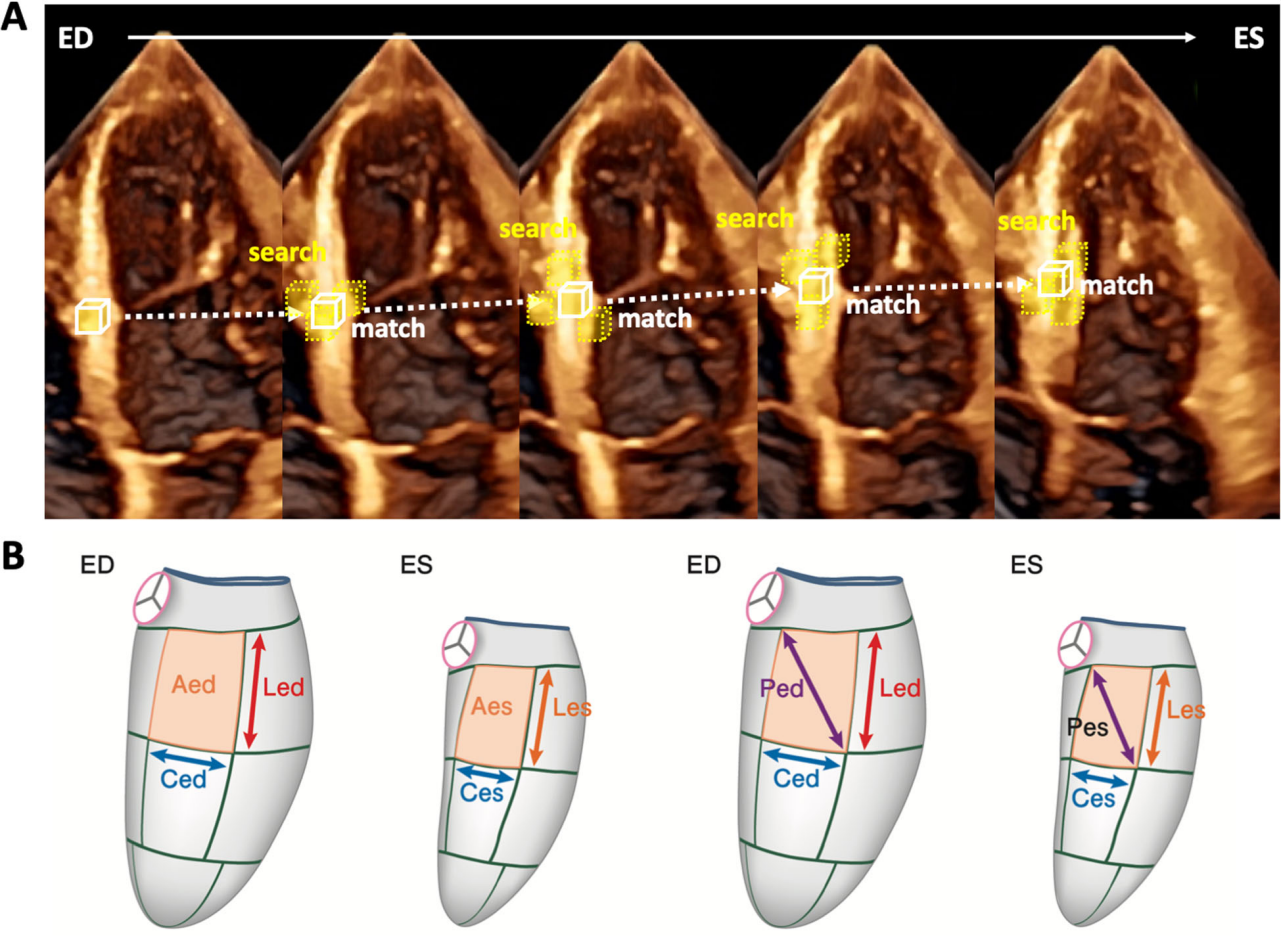
3D strain analysis. The upper panel illustrates the concept of block matching. The software searches for a cube in 3D space that is most similar to the cube in the previous frame (white cube). The same process is performed throughout one cardiac cycle, from which the software computes distance, time, and direction. Lower panels show calculation of area and principal strain. Ced(es) signifies circumferential distance at end-diastole (end-systole). Led(es) denotes longitudinal distance at end-diastole (end-systole). Aed(es) represents area at end-diastole (end-systole). Area strain is defined as Aed – Aes / Aed (%). Principal strain is defined as Ped -Pes / Ped (%).ED, end-diastole; ES, end-systole

**Fig. 2:**
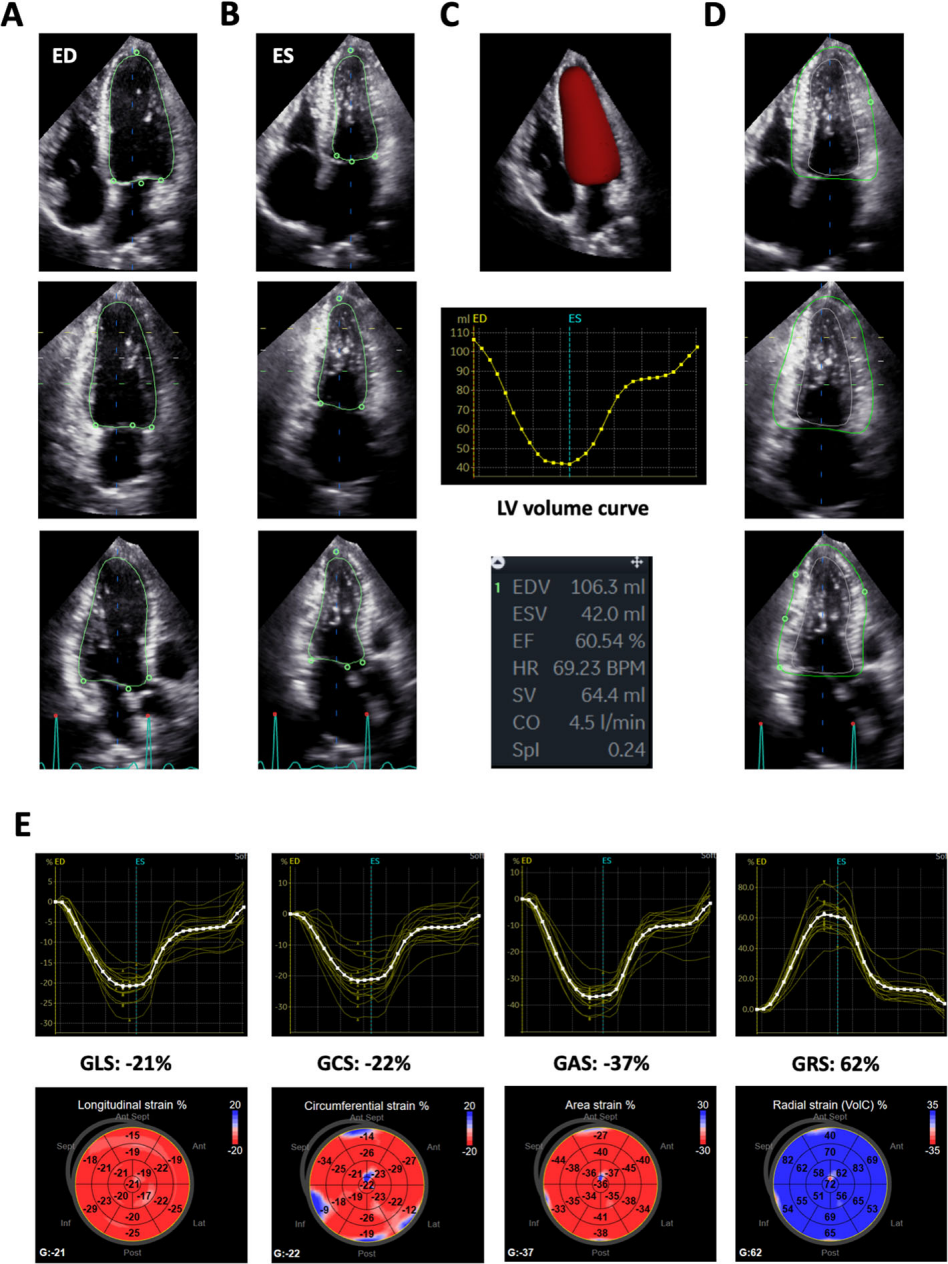
3D strain analysis using GE software in a healthy subject. Left ventricular endocardial border determination in apical 4-, 2-, and long-axis views at end-diastole (**a**) and end-systole (**b**). Green lines indicate the endocardial border. The software performed LV border determination during one cardiac cycle, and the generated LV volume curve, from which LV end-diastolic volume, end-systolic volume, and LV ejection fraction (LVEF) were calculated. A beutel of the left ventricle is also shown (**c**). Epicardial border determination with manual editing identified the region of interest for subsequent speckle tracking (**d**). 3D regional and global longitudinal strain curves, a circumferential strain curve, an area strain curve, a radial strain curve, and a bull’s eye map of corresponding regional strains (**e**), generated with 3D speckle tracking analysis. CO, cardiac output; ED, end-diastole; EDV, end-diastolic volume; EF, ejection fraction; ES, end-systole; ESV, end-systolic volume; HR, heart rate; GAS, global area strain; GCS, global circumferential strain; GLS, global longitudinal strain; GRS, global radial strain; Spl, sphericity index; SV, stroke volume

**Fig. 3:**
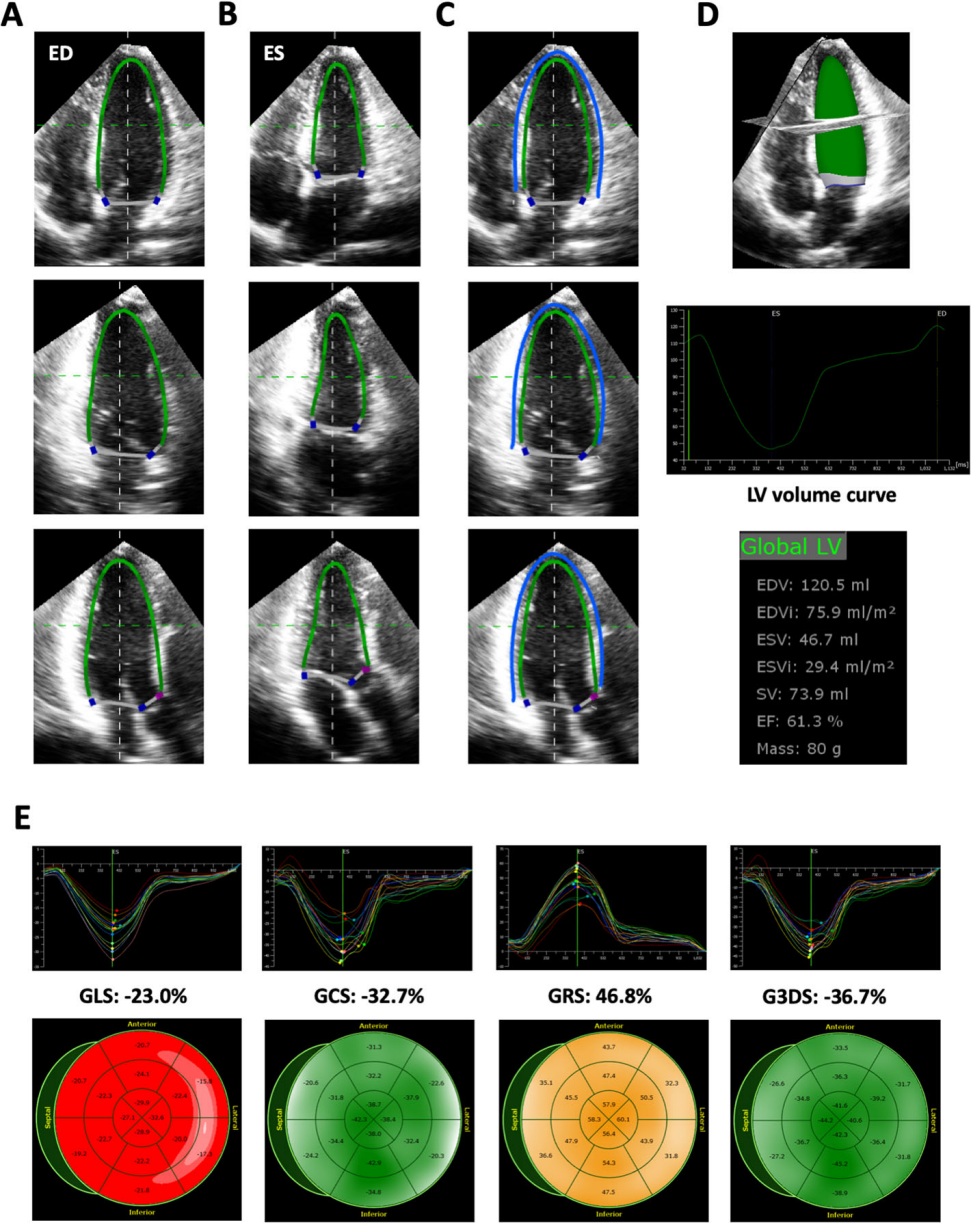
3D strain analysis using TomTec software in a healthy subject. Left ventricular endocardial border determination on the apical 4-, 2-, and long-axis views at end-diastole (**a**) and end-systole (**b**). Manual epicardial border determination resulting in LV mass analysis (**c**). Green lines indicate the endocardial border, and blue lines indicate the epicardial border. The software performed 3D speckle tracking analysis on the endocardial border, generating an LV volume curve, from which LV end-diastolic volume, end-systolic volume, and LV ejection fraction (LVEF) were calculated (**d**). The software also provided 3D regional and global longitudinal strain curves, a circumferential strain curve, a radial strain curve, and a 3D principal strain curve. Corresponding bull’s eye maps were also provided (**e**). ED(S)Vi, left ventricular end-diastolic (end-systolic) volume index; G3DS, global principal strain. Other abbreviations are the same as in Fig. 2

**Fig. 4:**
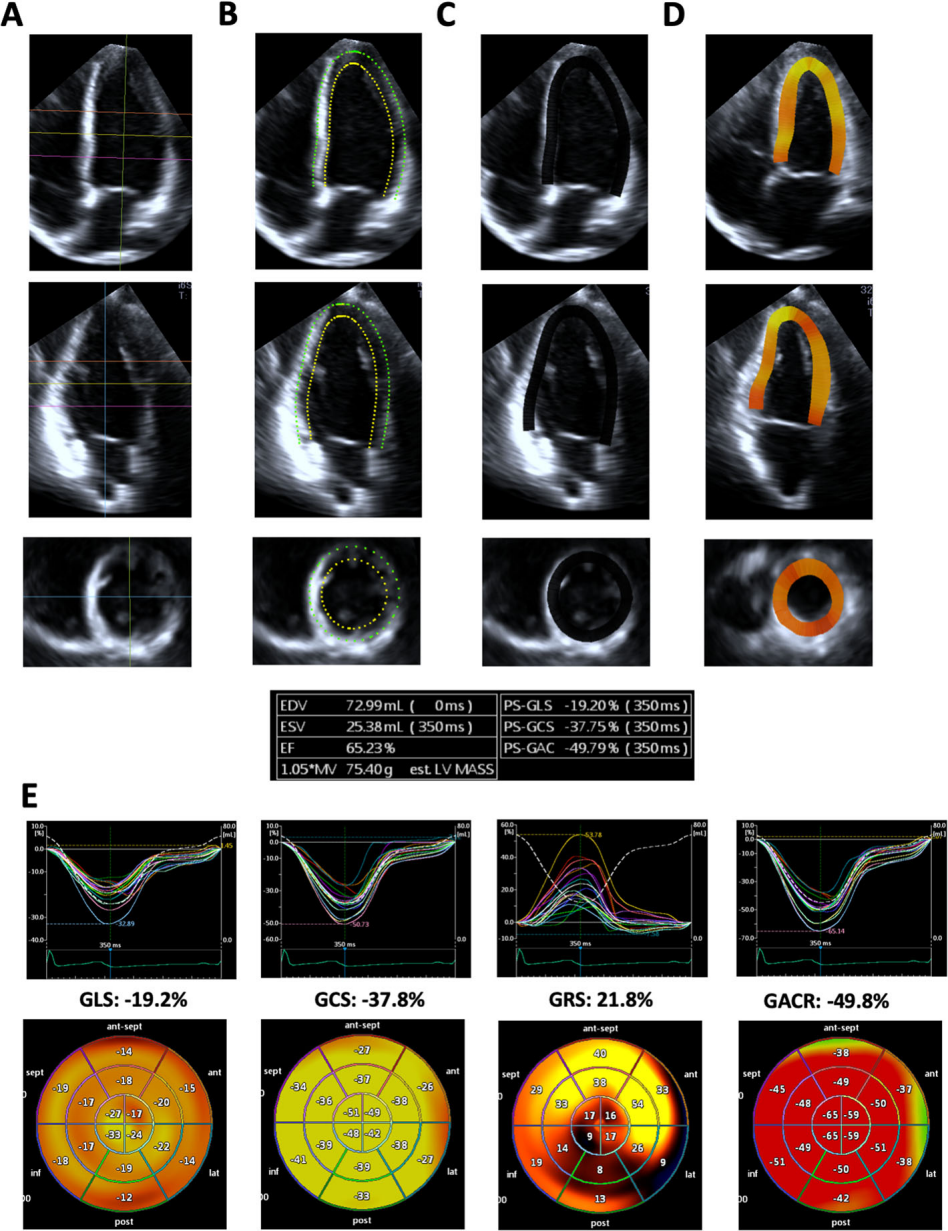
3D strain analysis using Canon software in a healthy subject. Apical and short axis views extracted from the 3DE dataset (**a**). The software automatically determined the region of interest (**b**), and performed 3D speckle tracking analysis, generating a color-coded strain map at end-diastole (**c**) and end-systole (**d**). In addition to LV volumes and LVEF, the software provided regional strain curves and bull’s eye map of longitudinal strain, circumferential strain, radial strain, and area change ratio (**e**). GACR, global area change ratio; MV, myocardial volume; PS, peak systolic. Other abbreviations are the same in Fig. 2

3D strain analysis starts by generating a region of interest (ROI), followed by segmentation of the left ventricle into a 16- or 17-segment model. The size of the ROI differs among 3D strain analytical software packages (LV subendocardial myocardium or the entire LV myocardium). Moreover, some software uses a library of various LV shapes from a large volume of patient data, and selects an appropriate LV shape to generate an ROI in each case (artificial intelligence). While 2D speckle tracking analysis pursues areas that contain specific natural acoustic markers, frame by frame within the ROI (pattern matching), 3D speckle tracking analysis tracks cubes with specific 3D patterns of acoustic markers within the ROI (block matching) to calculate global and regional 3D strain components (Fig. 1). Since the endocardial border is tracked throughout the cardiac cycle, the software also provides time domain LV volume curves from which the LV ejection fraction (LVEF) is calculated. In addition to longitudinal strain, circumferential strain, and radial strain, 3D strain software provides new deformation parameters, such as area strain and principal strain (Figs. 1, 2, 3 and 4). Area strain is determined as the percentage decrease in the size of endocardial (or mid-myocardial) surface area, defined by vectors of longitudinal and circumferential deformation at end-systole from its original area at end-diastole [6]. Principal strain represents the major direction and magnitude of the deformation in which no shear strain occurs. It reflects longitudinal, circumferential, and torsional deformation; therefore, it can represent dynamic 3D movements of the left ventricle [7].

It is important to note that each software system has its own proprietary tracking algorithm, with different analytical approaches. Although strain values should be 0 at the start and end of the cardiac cycle, this does not always happen in regional speckle tracking analysis. Some software rejects regional strain values if drift exceeds a certain level, but other vendors use drift compensation to force strain values to 0 at the end of the cardiac cycle. Radial strain is calculated as a change in transmural wall thickness during a cardiac cycle. Since the myocardium is incompressible, some vendors estimate radial strain from segmental area changes, assuming volume conservation [6]. Strain values may be represented as end-systolic strain or peak strain. These differences result in inter-vendor variability of 3D strain values that are significant, even when 3DE images are acquired from the same subjects [8].

### Characteristics of 2D and 3D strain

2D strain requires acquisition of multiple 2DE images, including three short-axis views to measure global circumferential strain (GCS) and global radial strain (GRS), and three apical views to measure global longitudinal strain (GLS), resulting in longer data acquisition times. For analysis of all strain components by 2DE, adequate quality of both parasternal and apical images is necessary. However, temporal and spatial resolution are higher, and feasibility is usually high. There have been several validation studies, and normal 2D global strain values have been reported from various large-scale studies [9–12]. Since 2D strain analysis is performed on a fixed 2DE cutting plane, some speckles or features may be lost in the 2DE imaging plane during a cardiac cycle, due to out-of-plane or twisting motion of the left ventricle [13].

In contrast, only a single apical acquisition is required for 3D strain analysis, resulting in shorter acquisition times and the opportunity to measure all 3D strain components from a single cardiac cycle. 3D strain measurements are not impacted by out-of-plane and twisting motion. However, 3D strain suffers from lower temporal and spatial resolution, which adversely affects tracking reliability. Multi-beat acquisition sometimes produces a stitching artifact between sub-volumes, which results in inaccurate speckle tracking analysis.

### Validation of 3D strain

Reliability and accuracy of 3D strain measurements have been validated in several studies (Table 1) [14–18]. First, the accuracy of regional longitudinal, circumferential, and radial strain measurements was determined in experimental studies under different loading conditions, using sonomicrometry data as a reference [14]. There is good agreement between 3D regional strain measurements and corresponding values acquired using sonomicrometry. The best results have been observed in circumferential strain. The same authors subsequently reported that endocardial area strain correlated strongly with sonomicrometry data [15]. Area strain values differed significantly with baseline, pharmacologic stress, and acute ischemia, whereas there were no differences in longitudinal and circumferential strains at baseline or with propranolol infusion or dobutamine infusion, indicating that area strain is a more sensitive parameter than longitudinal or circumferential strain for detecting subtle changes in LV deformation. Other authors applied 3D strain analysis in humans to validate its accuracy. However, it is important to note that there is no true “gold standard” for 3D myocardial mechanics [19]. Instead they employed surrogates, such as cardiac magnetic resonance myocardial tagging [16], feature tracking [18], or 2DE/3DE-derived LVEF [17].

**Table 1:** Validation studies for 3D strain

Experimental studies										
Author Year (Ref.#)	Vendor	Materials	Reference	3D regional LS		3D regional CS		3D regional RS		3D regional AS
Seo 2009 [14]	Toshiba	Sheep	Sonomicrometry	r = 0.89 *p* < 0.001		r = 0.90 *p* < 0.001		r = 0.84 *p* < 0.001		NA
Seo 2011 [15]	Toshiba	Sheep	Sonomicrometry	NA		NA		NA		r = 0.87 *p* < 0.001
Human studies										
Author Year (Ref.#)	Vendor	Subjects	Reference	3D GLS	3D GCS	3D GRS	3D GAS	2D GLS	2D GCS	2D GRS
Kleijn 2012 [16]	Toshiba	Volunteer	CMR tagging	NA	r = 0.80 p: NA	NA	NA	NA	NA	NA
Luis 2014 [17]	GE	Patient	2D LVEF	r = 0.74 *p* < 0.001	r = 0.89 *p* < 0.001	r = 0.86 *p* < 0.001	r = 0.87 *p* < 0.001	r = 0.86 *p* < 0.001	r = 0.82 *p* < 0.001	r = 0.67 *p* < 0.001
Luis 2014 [17]	GE	Patient	3D LVEF	r = 0.75 *p* < 0.001	r = 0.89 *p* < 0.001	r = 0.87 *p* < 0.001	r = 0.88 *p* < 0.001	r = 0.86 *p* < 0.001	r = 0.84 *p* < 0.001	r = 0.64 *p* < 0.001
Obokata 2016 [18]	TomTec	Patient	CMR feature tracking	r = 0.87 *p* < 0.001	r = 0.88 *p* < 0.001	r = 0.82 *p* < 0.001	NA	r = 0.83 *p* < 0.001	r = 0.90 *p* < 0.001	r = 0.69 *p* < 0.001

*AS* area strain, *CMR* cardiac magnetic resonance, *CS* circumferential strain, *GAS* global area strain, *GCS* global circumferential strain, *GLS* global longitudinal strain, *GRS* global radial strain, *LS* longitudinal strain, *LVEF* left ventricular ejection fraction, *NA* not available, *RS* radial strain

### Feasibility of 3D strain

The main impediment to acceptance of 3D strain is the lack of proof that it is truly superior to 2D strain for clinical use, beyond its theoretical advantages, resulting in its use mainly for research. A recent European Association of Cardiovascular Imaging (EACVI) survey from 96 echo laboratories in 22 European countries revealed that only 32% of European laboratories routinely use transthoracic 3DE, mainly due to limitations in image quality and operator dependency [20]. We performed a meta-analysis concerning the feasibility of 2D and 3D GLS in studies with sample sizes > 100 patients [21–29]. Feasibility of 3D GLS was 85% (95% confidence interval (CI): 79 to 89%). Corresponding values of 2D GLS were 91% (95% CI: 88 to 93%). If we compared feasibility of 3D GLS among different ethnicities, values were 91% (95% CI: 84 to 95%) in Asians, 81% (95% CI: 71 to 88%) in Europeans, and 73% (95% CI: 65 to 80%) in American patients. These results showed that feasibility of 3D GLS is lower than that of 2D GLS, and that feasibility is better in Asian patients than in American or European patients. However, there could be a selection bias, since some studies only selected patients with adequate 2DE image quality [23–25], and patients with poor 2DE image quality (the authors did not mention the number of these patients in the manuscripts) were excluded before strain analysis. Thus, actual feasibility of 2D/3D strain in consecutive series of patients could be much lower than the observed results. All published studies came from echocardiographic laboratories where physicians and sonographers were thoroughly familiar with 3DE data acquisition and analysis. Thus, the results may not be generally applicable.

### Direct comparisons of 2D and 3D strain values

**Fig. 5:**
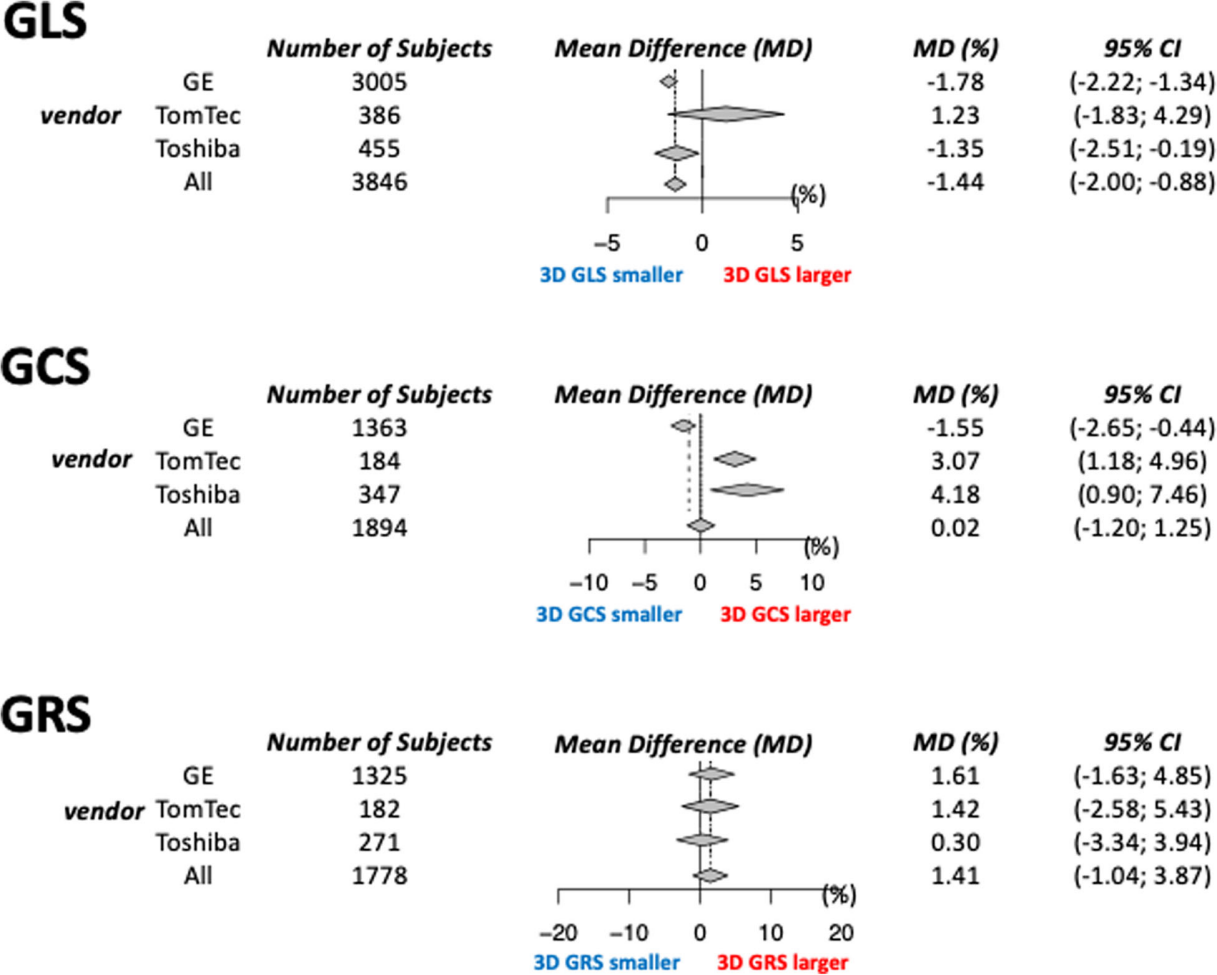
Direct comparison of 2D and 3D global strain. Data are presented according to each vendor and whole subjects. CI, confidence interval; MD, mean difference

We performed a meta-analysis to compare global 2D and 3D strain values using the same ultrasound vendor’s 2D and 3D strain software. We selected only publications in which 2D GCS/GRS were measured using three short-axis views, and 2D GLS was measured with three apical views. We found 3846 paired comparisons of GLS between 2DE and 3DE in 36 publications using one of the three vendors (GE Healthcare, TomTec Imaging Systems, Toshiba Medical Systems) [13, 17, 18, 21–23, 25–28, 30–55]. The mean value of 3D GLS was significantly lower than that of 2D GLS, with a mean bias of 1.4% (Fig. 5). The pooled mean value of 3D GLS for GE and Toshiba was significantly lower than that of 2D GLS. However, there were no significant differences when using TomTec software. Regarding GCS, there were no statistically significant differences between 3D GCS and 2D GCS in 1894 paired comparisons [13, 18, 23, 25, 26, 30, 35–37, 40–43, 48, 50, 56]. Vendor analysis showed that 3D GCS was significantly smaller than 2D GCS when GE analytical software was used, but 3D GCS was significantly larger than 2D GCS when we used Toshiba or TomTec software. Finally, there were no significant differences between 3D GRS and 2D GRS in 1778 paired comparisons from 14 studies [18, 23, 25, 26, 30, 36, 37, 40–43, 48, 56, 57]. Although software from all three vendors yielded no differences in GRS between the two techniques, results in each publication showed different trends, such that 2D GRS was larger than 3D GRS in some studies [25, 26, 42, 43] and 2D GRS was smaller than 3D GRS in others [23, 36, 37, 41, 56]. These results showed that differences in global strain between 2DE and 3DE vary according to the ultrasound vendor. Thus, 2D and 3D strain are not interchangeable, and we need vendor-dependent reference values of 3D strain.

### Reference values for 3D GLS

Truong et al. [58] recently performed meta-analysis to determine normal ranges of LV 3D GLS, 3D GCS, 3D GRS, and 3D GAS in 2346 subjects from 32 studies. The authors reported that the mean value of 3D GLS was 19.1%, ranging from 15.8 to 23.4% among the studies. However, the majority of selected studies had small sample sizes (*n* = 20–50), and the authors were forced to exclude one large study because median and interquartile range were presented in the manuscript, rather than means ± standard deviations [59]. When we searched publications in which at least 100 healthy subjects were evaluated, there were seven independent datasets from six publications (Table 2) [59–64]. Overall, the mean value of 3D GLS was 18.1% (95% CI: 16.1 to 20.1%). Mean values of 3D GLS ranged from 15.2 to 21.0% among the studies, and if we defined the lower limit of normal (LLN) as the mean – 1.96SD, LLN ranged from 11.1 to 15.9%. The mean value of 3D GLS in male subjects (*n* = 696) was 18.4% (95% CI: 16.5 to 20.3%) in four studies that addressed gender-based strain analysis [59, 60, 62, 63]. The corresponding value in female subjects (*n* = 818) was 19.8% (95% CI: 17.7 to 21.8%). It is interesting that 3D GLS in female subjects was consistently and significantly higher than in male subjects, irrespective of the software used for the analysis, with a mean bias of 1.3% (95% CI: 0.9 to 1.7%). This finding suggests the need for establishment of gender-dependent reference values. Finally, as in a previous 2D GLS study [12], there were inter-vendor differences in 3D GLS reference values. The lowest values were observed when Toshiba software (15.4 ± 1.4%, *n* = 634) was used, followed by GE software (18.7 ± 2.6%, *n* = 265). The highest values were obtained with TomTec software (20.6 ± 3.0%, *n* = 946). This is partly because ultrasound vendors use different ROIs for speckle tracking (both TomTec and Toshiba software use subendocardial tracking [14], but GE software uses full myocardial tracking). Different 3D dataset characteristics in each vendor is another cause of discrepancy [65]. The World Alliance Societies of Echocardiography Normal Values Study plans to address whether racial differences in 3D strain are observed [11].

**Table 2:** Reference range of 3D global longitudinal strain from six large studies

Author year (Ref.#)	n	Race	Vendor	Tracking	Mean ± SD	LLN (Overall) (mean-1.96SD)	Mean ± SD male	Mean ± SD female	P (male vs. female)	LLN (Male) (mean-1.96SD)	LLN (Female) (mean-1.96SD)
Kaku 2014 [59]	241	Japanese/American	TomTec	Subendocardial	19.6 ± 3.1%	13.5%	18.7 ± 2.8%	20.6 ± 3.1%	< 0.001	13.2%	14.5%
Xia 2014 [60]	153	Chinese	Toshiba	Full myocardial	15.2 ± 0.8%	13.6%	NA	NA	NA	NA	NA
Muraru 2014 [58]	265	European	GE	Full myocardial	18.7 ± 2.6%	13.5%	17.8 ± 2.4%	19.4 ± 2.6%	< 0.001	13.0%	14.2%
Muraru 2014 [58]	265	European	TomTec	Subendocardial	20.4 ± 3.8%	12.8%	19.5 ± 3.8%	21.1 ± 3.6%	< 0.001	11.9%	13.9%
Kleijn 2015 [61]	303	European/American	Toshiba	Full myocardial	15.9 ± 2.4%	11.2%	15.5 ± 2.4%	16.3 ± 2.3%	0.003	10.8%	11.8%
Bernard 2017 [62]	440	European	TomTec	Subendocardial	21.0 ± 2.6%	15.9%	20.4 ± 2.7%	21.4 ± 2.4%	< 0.001	15.1%	16.7%
Kovacs 2019 [63]	178	European	Toshiba	Full myocardial	16.1 ± 2.5%	11.1%	NA	NA	NA	NA	NA

*LLN* lower limit of normal, *NA* not available, *SD* standard deviation

Other abbreviations are the same in Table 1

### Clinical applications of 3D strain

Since clinical applications of 3D strain are still limited, we focus here on its usefulness in several clinical scenarios.

#### Ischemic heart disease

Even in patients who had received successful primary percutaneous coronary intervention after acute myocardial infarction, when LV adverse remodeling occurs, it is associated with poor outcomes; thus, for reliable, accurate assessment, it is of paramount importance to stratify high risk patients after the intervention. Infarct size negatively affects LV function; thus, it is a major determinant of LV adverse remodeling. 2D and 3D deformation parameters, which reflect regional and global LV function, may reliably evaluate infarct size and transmural extent of myocardial infarction. Several studies have demonstrated the clinical usefulness of 3D strain analysis in patients with acute or recent myocardial infarction, who underwent primary percutaneous coronary intervention [28, 31, 34, 50, 66–70]. Some studies addressed the utility of 3D strain for predicting LV adverse remodeling (defined as an increase in LV end-diastolic volume ≥ 15% or 20%) or LV functional recovery (defined as an improvement ≥5% of LVEF) during the chronic phase (Table 3) [28, 31, 34, 50, 66–69]. 3D global strain analysis was possible in > 80% of study subjects. All studies revealed that 3D global strain, especially 3D GLS and 3D GAS, predict LV adverse remodeling with moderate accuracy (57 to 75%). Only one study compared predictive values of 2D and 3D global strain. It showed that 3D GLS was a significantly better predictor of LV adverse remodeling than 2D GLS, 3D GAS, or 3D GRS [28].

**Table 3:** Usefulness of 3D strain in patients with myocardial infarction

Author Year (Ref.#)	n	Patients	Vendor	Feasibility		Remarks				
LV remodeling										
Abate 2012 [64]	153	Acute STEMI	GE	96% (patients)		1) Regional 3D LS of 11.1% had a > 90% sensitivity and specificity for predicting improvement of LV regional function. 2) 3D GLS had an incremental value over clinical and standard echocardiography parameters for predicting improvement of LVEF (> 5%).				
Li 2012 [65]	61	Recent NSTEMI	Toshiba	80% (patients)		1) Regional AS > 23% at baseline had a 75% sensitivity and a 76% specificity for predicting improvement of LV regional function. 2) 3D GAS ≤32% after PCI predicted LV adverse remodeling with 86% sensitivity and 68% specificity.				
Sugano 2017 [49]	71	Acute STEMI	Toshiba	96% (patients)		1) 3D GCS < 23% had an 84% sensitivity and a 74% specificity for predicting LV adverse remodeling. 2) 3D GAS < 31% had an 84% sensitivity and a 58% specificity for predicting LV adverse remodeling.				
Xu 2017 [27]	110	Acute STEMI	GE	91% (patients)		1) 2D GLS, 3D GLS, 3D GAS, and 3D GRS were independent predictors of LV adverse remodeling. 2) 3D GLS < 12.6% had a 92% sensitivity and a 60% specificity for predicting LV adverse remodeling. 3) 3D GAS < 24.2% had a 92% sensitivity and a 46% specificity for predicting LV adverse remodeling. 4) AUC of 3D GLS (0.82) was significantly higher than that of 2D GLS (0.72), 3D GAS (0.68) and 3D GRS (0.68) for predicting LV adverse remodeling.				
Transmurality of MI										
Hayat 2012 [30]	25	OMI	Toshiba	76% (patients) 96% (segments)		1) 2D LS and CS and all 3D regional strains were significantly different among segments derived from control subjects, those with non-transmural MI, and those with transmural MI.				
Thorstensen 2013 [33]	58	RMI	GE	62% (patients) 71% (segments)		1) All 3D regional strains were significantly different among segments with no MI, those with non-transmural MI, and those with transmural MI. 2) All 3D regional strains predicted transmural MI (AUC: 0.73–0.87). 3) 2D GLS had a higher AUC (0.88) for the prediction of transmural MI than 3DGLS (0.73, *p* < 0.05).				
Zhu 2014 [66]	26	AMI	Toshiba	Not described		1. Regional 3D LS and 3D CS discriminated among segments with no MI, those with non-transmural MI, and those with transmural MI.				
Aly 2016 [67]	82	LV dysfunction	Toshiba	88% (segments)		1. Regional 3D CS and 3D AS discriminated among segments with no MI, those with non-transmural MI, and those with transmural MI. 2. Regional 3D LS discriminated between segments with non-transmural MI and those with transmural MI. 3. Included 11 patients with non-ischemic LV dysfunction.				
Sugano 2017 [49]	71	Acute STEMI	Toshiba	95% (segments)		1. Regional 2D CS, 3D CS, and 3D AS discriminated among segments with no MI, those with non-transmural MI, and those with transmural MI. 2. Regional 2D LS failed to differentiate between non-transmural MI and transmural MI. 3D LS failed to differentiate between no MI and non-transmural MI.				
Infarct size										
Author Year (Ref.#)	n	Patients	Vendor	Correlation of infarction size						remarks
				2D GLS	3D GLS	3D GCS	3D GRS	3DS	3D AS	
Hayat 2012 [30]	25	OMI	Toshiba	NA	r = 0.45	r = 0.47	r = 0.07	r = 0.10	r = 0.49	
Thorstensen 2013 [33]	58	RMI	GE	r = 0.67	r = 0.42	r = 0.47	r = 0.48	r = 0.52	r = 0.50	2DGLS was more closely correlated with infarct size than 3DGLS.
Zhu 2014 [66]	26	AMI	Toshiba	NA	r = 0.86	r = 0.81	r = 0.71	NA	NA	
Aly 2016 [67]	71	ICM	Toshiba	NA	r = 0.29	r = 0.32	r = 0.08	r = 0.29	r = 0.39	

*AMI* acute myocardial infarction, *AUC* area under the curve, *AS* area strain, *CS* circumferential strain, *LS* longitudinal strain, *GAS* global area strain, *GCS* global circumferential strain, *GLS* global longitudinal strain, *GRS* global radial strain, *MI* myocardial infarction, *NA* not available, *OMI* old myocardial infraction, *RMI* recent myocardial infraction, *STEMI* ST elevation myocardial infarction

Since endocardial fibers are oriented longitudinally, and mid-myocardial fibers run circumferentially, preferential reduction of longitudinal strain and preserved circumferential strain could reflect the presence of subendocardial myocardial infarction, and reduction of both longitudinal and circumferential strains may represent transmural myocardial infarction. Other studies investigated the diagnostic value of 3D strain measurements for evaluating the transmurality of infarction, which was verified using cardiac magnetic resonance with late gadolinium enhancement (LGE) (subendocardial infarction, 0% to ≤50%; transmural infarction, > 50% of the transmural extent of LGE) [31, 34, 50, 68, 69]. In contrast to the aforementioned theory, all 3D regional strain measurements differed significantly among segments with no myocardial infarction, segments with non-transmural infarction, and segments with transmural infarction. In addition, none of the studies unequivocally demonstrated that 3D regional strain is superior to 2D regional strain for evaluating transmurality of myocardial infarction.

The potential utility of 3D strain to determine myocardial infarct size has been also reported [31, 34, 68, 69]. Overall, there were modest correlations between 3D GLS/ 3D GCS and infarct size assessed by LGE using cardiac magnetic resonance imaging. A meta-analysis revealed that the r value for the correlation between 3D GLS and the percentage of myocardial infarction size in four studies [31, 34, 68, 69] was only 0.55 (95% CI: 0.19 to 0.78) with notable heterogeneity [70]. Pooled r values further decreased to 0.38 (95% CI: 0.22 to 0.54) when one study that was a potential cause of heterogeneity was excluded from the analysis.

These results suggest that 3D global strain analysis may be useful to predict LV adverse remodeling. However, their diagnostic accuracy is modest at best. 3D regional strain measurement is not sufficiently sensitive to discriminate between subendocardial infarction and transmural infarction. 3D global strain values are not useful to determine infarct size. There are also no consistent findings to suggest that 3D strain is more useful than 2D strain. Further large-scale studies are required to determine whether 3D strain has some added value over 2D strain for evaluating patients with myocardial infarction after coronary intervention.

#### Cardio-oncology

As the development of tumor-targeted anticancer drugs allows cancer patients to live longer, cancer therapy-related cardiac dysfunction and/or heart failure is becoming a major issue [71]. Although cardiac dysfunction in patients undergoing cancer treatment is complex with respect to treatment regimen, cardiotoxic anticancer drugs, age, and comorbidities, detection of subclinical myocardial dysfunction facilitates timely intervention and reduces the risk of adverse outcomes. 2D strain has proven useful to detect subclinical LV dysfunction in cancer patients [72]. Several studies have attempted to determine the clinical usefulness of 3D strain in cancer patients who had been treated with cardiotoxic anticancer drugs [27, 40, 54, 73–76]. All but one study showed a significant reduction of 3D global strain during and after cardiotoxic anticancer drugs, compared with baseline (Table 4). A significant reduction of 3D global strain was already observed at a timepoint when 2D LVEF and/or 2D global strains were still normal in some studies [73, 74]. Another study revealed that 3D LVEF and 3D global strains were associated with concurrent and subsequent changes in 2D LVEF [75]. However, there were no consistent findings that a specific 3D strain component is most robust for detecting subtle LV dysfunction. There were also very few articles that validated added value of 3D strain over 2D strain for predicting LV dysfunction [75]. Thus, 3D strain can predict subclinical LV dysfunction, but data were insufficient to conclude whether 3D strain is better than 2D strain.

**Table 4:** Summary of studies using 3D strain in cancer patients who had received cardiotoxic drugs

Author Year (Ref.#)	Type of cancer	n	Treatment	Echo timing	Feasibility	CTRCD (%)	Pre-echo	Post-echo	Remarks
Mornos 2014 [71]	various	79	Anthracycline (100%)	Before and at 12 and 36 weeks	3D STE: 75%	14%	3D GLS: 19.4 ± 2.3% 3D GCS: 21.4 ± 1.7% 3D GRS: 42.4 ± 5.3%	17.5 ± 2.4%* 20.9 ± 1.7% 37.6 ± 5.4%*	Δ3D GLS was an independent predictor for future CTRCD. Δ3D GLS of 13.7% had an 88% sensitivity and a 71% specificity for CTRCD.
Tarr 2015 [39]	various	25	Anthracycline (28%)	Before and 3 months	2D STE: 100% 3D STE: 100%	NA	2D GLS: 15.0 ± 4.2% 2D GRS: 28.0 ± 10.6% 3D GLS: 13.0 ± 2.6% 3D GCS: 21.0 ± 4.5% 3D GRS: 28.0 ± 12.8%	14.0 ± 4.6%* 21.0 ± 11.5%* 12.0 ± 2.2% 22.0 ± 4.7% 26.0 ± 14.5%	3D global strains did not show a significant decrease after therapy.
Santoro 2017 [26]	breast	100	Anthracycline (100%)	Before and at 4 months	2D STE: 91% /90% 3D vol.: 88% /67% 3D STE: 84% /60%	NA	2D GLS: 22.2 ± 2.3% 3D LVEF: 62 ± 7% 3D GLS: 17.6 ± 3.2% 3D GCS: 16.8 ± 2.8% 3D GAS: 30.2 ± 4.5% 3D GRS: 47.4 ± 9.2%	20.1 ± 6.6%* 60 ± 7%* 16.2 ± 3.5%* 15.2 ± 2.9%* 27.5 ± 5.4%* 43.1 ± 10.7%	% reduction of 2D GLS > 15% was observed in 17 patients (17%). 3D-derived LVEF decreased < 50% in 4 out of 67 patients (6%).
Song 2017 [72]	lymphoma	89	Anthracycline (100%)	Before, at 3 weeks, and at the end of Tx.	2D STE: 93% /93%/93% 3D STE: 93% /93%/93%	NA	2D LVEF: 70 ± 3% 2D GLS: 21.5 ± 2.5% 3D GLS: 21.8 ± 2.9% 3D GCS: 29.9 ± 4.4%	69 ± 3% 20.7 ± 2.1% 27.5 ± 4.5%* 27.3 ± 5.0%*	2D GLS and LVEF did not change, but 3D GLS and 3D GCS were significantly reduced 3 weeks after therapy.
Zhang 2018 [73]	breast	142	Anthracycline (100%) Trastuzumab (9%)	Before and annually	3D STE: 94%	NA	3D LVEF: 56.8% 3D GLS:16.8% 3D GCS: 27.3% 3D principal strain: 29.9%	51.5%* 15.3%* 24.2%* 26.0%*	3D LVEF, 3D GLS, 3D GCS, and 3D principal strain were associated with concurrent and subsequent changes in systolic function.
Chen 2019 [74]	breast	83	Anthracycline (100%)	Before, during, and after Tx.	3D STE:100%	NA	3D GLS 18.1 ± 2.2% 3D GCS 18.7 ± 2.6% 3D GAS 34.1 ± 2.8% 3D GRS 44.9 ± 5.2%	14.9 ± 2.5%* 15.7 ± 0.3%* 23.9 ± 2.6%* 43.3 ± 4.9%	There was a significant correlation between 3D GAS and the culminating dose of anthracycline (r = 0.77).
Cruz 2019 [53]	breast	105	Anthracycline (100%) Trastuzumab (52%)	Before and during Tx.	2D STE: 100% (patients)/ 96% (segments) 3D STE: 100% (patients)/ 94% (segments)	23%	2D LVEF: 66 ± 8% 2D GLS: 21.1 ± 3.0% 3D LVEF: 62 ± 6% 3D GLS: 15.6 ± 3.4% 3D GCS: 14.0 ± 4.0% 3D GAS: 27.0 ± 8.5% 3D GRS: 42.0 ± 17.0%	58 ± 11%* 18.8 ± 3.1%* 54 ± 9%* 10.9 ± 4.1%* 11.0 ± 5.0%* 20.0 ± 9.0%* 28.5 ± 17.5%*	Percent reduction of 3D GCS (cut-off value of 34.2%) and that of 3D GRS (34.5%) predicted CTRCT with 70% diagnostic accuracy.

CTRCD, cancer therapy related cardiac dysfunction; STE, speckle tracking echocardiography; Tx., therapy

Other abbreviations are the same in Table 1

*p < 0.05 versus baseline

Acquisition of good-quality echocardiographic images is a potential concern in breast cancer patients because patients often receive mastectomies and radiation therapy of the chest. A meta-analysis from seven studies (Table 4) revealed that feasibility of 3D strain measurements before and after treatment were 93% (95% CI: 85 to 97%) and 92% (95% CI:80 to 97%), respectively. However, it is worth noting that patients with bad image quality were excluded in some studies [40, 54]. In this regard, the results from Santoro and colleagues may represent the current feasibility of 2D/3D strain measurements in prospectively enrolled breast cancer patients [27]. This study also showed that 2D GLS was better than 3D global strain for evaluating treatment course in breast cancer patients due to high feasibility and reasonable detection of subclinical LV dysfunction.

Only two studies investigated the prognostic value of 3D global strain to predict cancer therapy-related cardiac dysfunction and/or heart failure with moderate accuracy (70 and 73%) [54, 73]. Thus, further studies are required to verify whether 3D strain is better than 2D strain to predict outcomes. It is also necessary to investigate whether patient management guided by 3D LVEF and/or 3D strain is better than that guided by 2D LVEF and/or 2D strain to prevent heart failure in cancer patients.

#### Subclinical LV dysfunction

LV longitudinal functional impairment in patients with preserved LVEF is the first component that has been demonstrated using 2D strain analysis [1, 2]. 3D strain analysis has been performed to verify this concept in asymptomatic patients with normal LVEF who had comorbidities (hypertension, diabetes or collagen disease) [6, 36, 37, 44, 49, 52, 56, 77]. Pooled analysis revealed that both 2D and 3D GLS in patients were consistently and significantly depressed compared with control subjects (Supplementary Figure 1). Thus, both 2D and 3D strain can detect subclinical LV dysfunction.

#### Left ventricular hypertrophy

LV hypertrophy (LVH) has been associated with increased cardiovascular death and all causes of death, independent of traditional risk factors [78]. The potential usefulness of 3D strain in patients with LVH is summarized in Table 5 [39, 47, 53, 79–83]. In patients with hypertension, Tadic et al. demonstrated that 2D and 3D global strain values differed among five types of LV geometry showing that the worst LV deformation parameters were observed in concentric LVH and dilated LVH, even after adjusting anthropometric and hemodynamic parameters [39].

**Table 5:** Summary of studies using 3D strain in patients with LV hypertrophy

Author Year (Ref #)	Type of disease (number)	Purpose	Remarks
Baccouche 2012 [77]	CA (*n* = 12) / HCM (*n* = 12)	To differentiate two pathologies.	1) Basal LS, CS and RS were significantly reduced in patients with CA compared with HCM. 2) Regional strain values were irreversibly correlated with LGE, and the best correlation was observed between RS and LGE (r = −0.82)
Aly 2014 [78]	HCM mutation carriers (*n* = 23) / HCM (*n* = 28) / control (*n* = 29)	To detect early changes in myocardial mechanics in HCM mutations.	1) There were no significant differences in 3D global/regional strains between HCM mutations and control subjects. 2) 3D global/regional LS and AS were significantly impaired in HCM compared with HCM mutations. 3) 3D GCS and 3DGRS were not different between HCM and HCM mutations.
Tadic 2015 [38]	HT with normal LV geometry (*n* = 85) / concentric LV remodeling (*n* = 28) / eccentric nondilated LVH (42) / concentric LVH (*n* = 30) / dilated and concentric-dilated LVH (*n* = 12)	To investigate LV mechanics in HT with different geometric patterns	1) 2D and 3D global strains decreased normal geometry, followed by concentric remodeling, eccentric nondilated LVH, concentric LVH, dilated LVH and concentric dilated LVH. 2) Reduced 2D and 3D strains were associated with concentric and dilated LVH patterns independent of demographic and clinical parameters.
Urbano-Moral 2015 [79]	AL amyloidosis (*n* = 40)	To detect cardiac involvement.	1) 3D GLS and GCS were significantly lower in patients with cardiac involvement than those without. 2) Prominent reduction of LS/CS was observed in the basal myocardium.
Voilliot 2015 [80]	HCM (*n* = 40) / control (*n* = 53)	To assess impact of hypertrophy on strains.	Compared to control subjects, 1) 3D GLS, GAS, and GRS were significantly lower in HCM patients. 2) No significant differences in 3D GCS were noted. 3) 3D regional LS/AS was significantly depressed irrespective to the degree of hypertrophy. 4) 3D regional CS was higher in no or mildly hypertrophied segments.
Ternacle 2017 [52]	Athlete with moderate LVH (*n* = 25) / Athlete without LVH (*n* = 25) / HCM (*n* = 25) / control (*n* = 25)	To differentiate patients with HCM from athletes with moderate LVH.	1) 2D GLS and 3D GLS were significantly lower in HCM than athletes with moderate LVH. 2) 2D LV dyssynchrony index (SD of time to peak LS in 16 segment model) had a highest AUC for identifying HCM in the presence of moderate LVH. 3) 3D GCS was not different between the two groups.
Cho 2017 [46]	Severe AS with normal LVEF (≥ 55%, *n* = 45) / control (*n* = 18)	To evaluate early myocardial dysfunction	1) 2D GLS and 3D GLS were significantly impaired in severe AS patients with increased LV wall thickness compared with normal LV wall thickness. 2) 3D GCS, GAS, and GRS did not show any differences between the two groups.
Pradel 2019 [81]	AL amyloidosis (*n* = 58) / control (*n* = 21)	To detect LV dysfunction.	1) There were no significant differences in 3D LVEF and 3D global strains between Mayo Clinic Stage I AL amyloidosis and control subjects. 2) 3D LVEF and 3D global strains decreased according to the advanced Mayo Clinic stage.

*AS* aortic stenosis, *CA* cardiac amyloidosis, *HCM* hypertrophic cardiomyopathy, *HT* hypertension, *LGE* late gadolinium enhancement, *LVH* left ventricular hypertrophy, *SD* standard deviation

Other abbreviations are the same in Table 1

Both 3D GLS and GAS were consistently impaired irrespective of the severity of LVH, but 3D GCS was preserved or even enhanced in patients with hypertrophic cardiomyopathy [53, 80, 82]. These results indicate that preservation of 3D GCS may be a compensatory mechanism for maintaining LVEF.

**Fig. 6:**
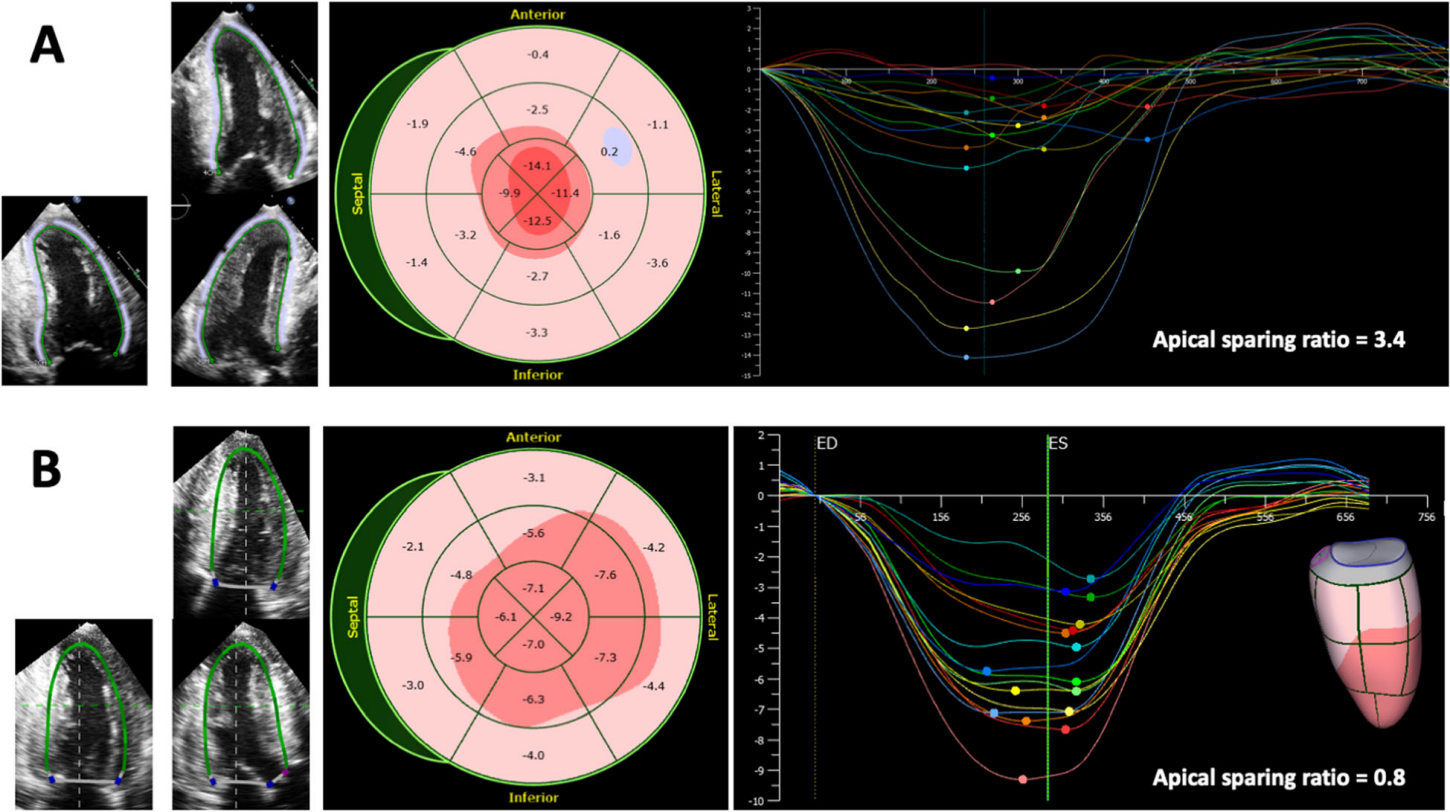
2D/3D strain analysis in a patient with cardiac amyloidosis. Upper panels show three 2D apical views, a bull’s eye plot of regional longitudinal strain (LS) and corresponding LS curves. Note the preservation of apical LS (apical sparing) with an apical sparing ratio of 3.4. Apical sparing ratio is defined as the average apical LS divided by (the average basal LS + the average middle LS). Lower panels show three 2D apical views extracted from the 3D dataset, a bull’s eye plot of regional LS, and corresponding curves in the same patient. Note the relative preservation of apical LS with an apical sparing ratio of 0.8

3D strains are also reportedly impaired in patients with cardiac amyloidosis [79, 83]. Regional assessment of strain values has been reported in only one study [79]. Compared to hypertrophic cardiomyopathy, basal radial strain was remarkably reduced, but apical radial strain was preserved in patients with cardiac amyloidosis, and the findings were compatible to the apical sparing pattern observed using 2D longitudinal strain [84] (Fig. 6). Finally, there are still not enough data to validate whether 3D strain has added value over 2D strain in patients with LVH.

#### Valvular heart disease

3D strain has been assessed in patients with valvular heart disease [26, 41, 47, 85–88]. Several authors analyzed 3D strain before and after transcatheter aortic valve replacement (TAVR) or MitraClip [41, 85, 87]. 2D and 3D GLS were significantly improved 6 months after TAVR in patients with severe aortic stenosis, and this beneficial trend was more obvious in patients whose baseline LVEF was more impaired, which reflects the presence of afterload mismatch [85]. All 3D global strains except 3D GRS were improved significantly 6 months after the MitraClip in patients with more than moderate mitral regurgitation [41]. The authors also noted that right ventricular dysfunction at baseline was associated with poor improvement of 3D global strains. The other study showed that 3D LV volumes, LVEF, and 2D GLS were not improved, but 3D GLS was improved 1 month after the MitraClip [87]. An impairment of 3D GLS with compensated augmentation of 3D GCS has been reported in asymptomatic patients with moderate or severe aortic regurgitation [88]. This compensating circumferential function could result in normal LVEF, a finding also reported in patients with hypertrophic cardiomyopathy [53, 80, 82].

#### Cardiac resynchronization therapy

Although echocardiographic assessment of LV mechanical dyssynchrony was expected to assist selection of optimal candidates for cardiac resynchronization therapy (CRT), because of the negative outcome of the PROSPECT trial, echocardiography was not recommended by current guidelines [89]. Several investigators have used 3D strain to evaluate LV dyssynchrony to predict CRT responders [90–94]. The authors measured time from onset of the QRS complex to peak strain, and determined the earliest and latest activation sites or created a new dyssynchrony index. However, it should be noted that time to peak strain does not reflect time to onset of mechanical contraction.

**Fig. 7:**
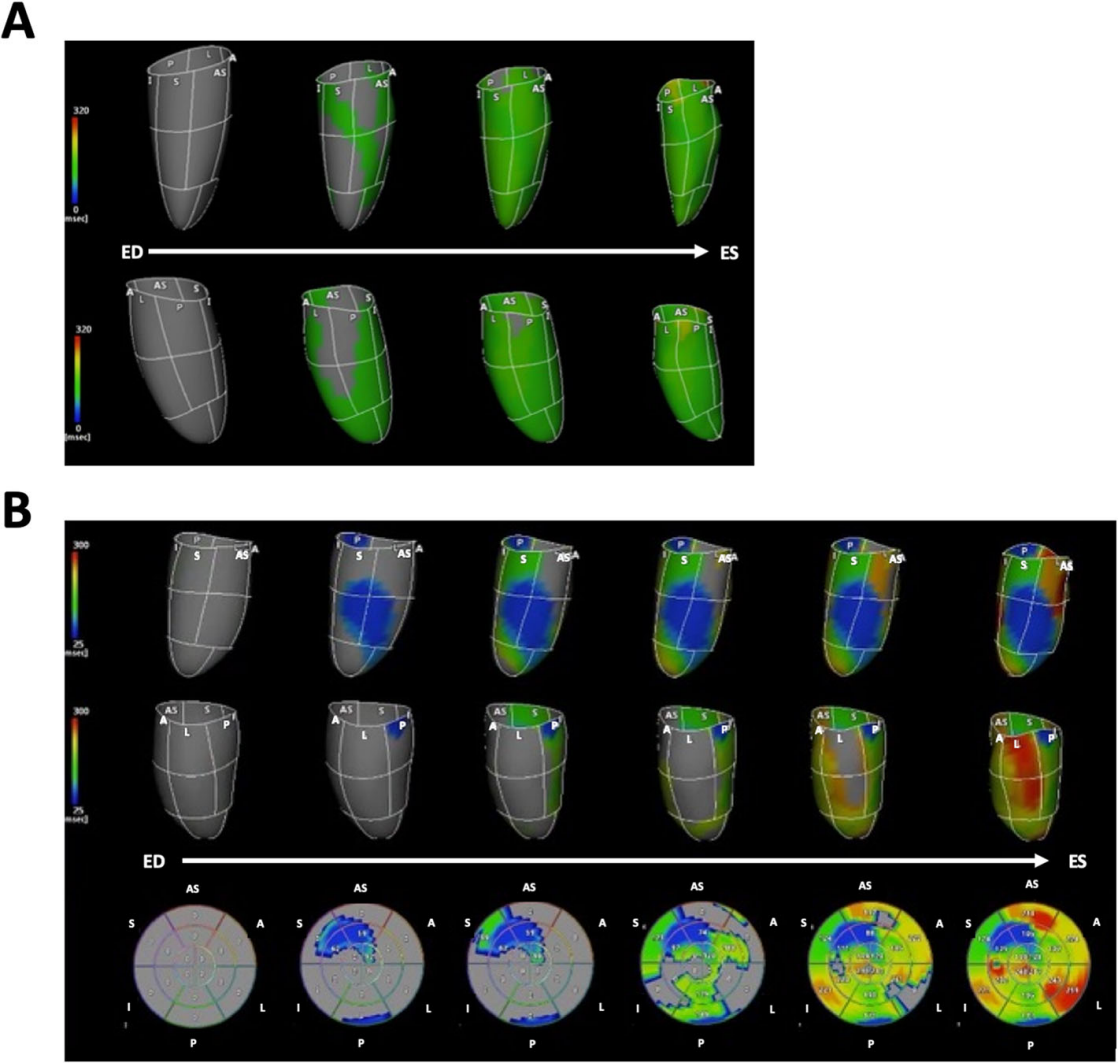
Activation mapping. **a**: Propagation images in a normal subject by activation imaging with 3D speckle tracking echocardiography. The upper panel shows propagation of myocardial contraction viewed from the left ventricular (LV) septal side from end-diastole (ED) to end-systole (ES), and the lower panel shows it viewed from the LV free wall. The color bar refers to a color-coded time scale that corresponds to the timing of the onset of regional contractions. In this case, the range is set from 0 ms (dark blue) to 320 ms (red) via green and yellow. The onset of myocardial contraction in the entire left ventricle is almost synchronized, reflecting light green colored area spreading rapidly. **b**: Propagation images in a patient with left bundle branch block. The upper panel shows propagation of LV myocardial contraction viewed from the septal side, and the middle panel shows it viewed from the LV free wall. The lower panel is a bull’s eye map displaying the spread of contraction. The upper panel shows early contraction in the septum colored in blue, which is earlier than septal contraction timing in normal subjects, and corresponds to “septal flash” during the pre-ejection period. The propagation of septal contraction is blocked at the anterior wall, but propagates toward the apex. The delayed propagation from the apex to the lateral wall is shown in a red-colored area in the basal to mid lateral wall, characterizing U-shaped propagation. A, anterior wall; AS, anterior septum; I, inferior wall; L, lateral wall; P, posterior wall; S, septal wall

Seo et al. developed an activation imaging system using 3D area strain analysis which sought to quantify time from the QRS complex to the onset of regional deformation, and its reliability and accuracy has been validated against 3D voltage-mapping systems [95]. Activation imaging allows detection of the earliest activation site, subsequent propagation sequence, and latest activation. The same authors also demonstrated that the presence of a U-shaped propagation pattern, which was characterized as activation propagated from the mid septum, followed by the apex to the lateral or posterior wall, had a sensitivity of 88% and a specificity of 95% for predicting CRT volume responder (≥15% reduction of LV end-systolic volume at 6 months after CRT) [96] (Fig. 7). A U-shaped propagation pattern was also associated with good outcomes after adjusting left bundle branch block or LV end-diastolic volume. Thus, activation imaging with 3D speckle tracking analysis has potential to visualize propagation of LV regional activation and to provide additional value for predicting outcomes in patients who are referred for CRT.

#### Prognostic value

Several studies investigated the prognostic value of 3D strain in myocardial infarction, hemodialysis, valvular heart disease, and a diverse variety of subjects [24, 26, 48, 86, 97–100] (Table 6). These studies all showed that 3D global strains were impaired in patients who had cardiac events, and reduction of 3D global strains was associated with poor outcomes. Reduction of 2D and 3D strains was attributed to myocardial fibrosis or inflammation [101]. Three of eight studies directly compared prognostic values between 2D GLS and 3D GLS, and all three studies showed that 3D GLS had superior prognostic value over 2D GLS [26, 99, 100]. Further studies are needed to validate whether 3D GLS is distinctly superior to 2D GLS for predicting future outcomes in specific cardiovascular pathologies.

**Table 6:** Prognostic values of 3D strain

Author Year (Ref. #)	n	Etiology	2DSTE	3DSTE	Events	Remarks
Chang 2014 [23]	200	Diverse	Not described	Toshiba	HF hospitalization or CD (*n* = 32)	1) All 3D global strains were associated with outcomes. 2) 3D GLS and 3D GRS had an incremental value over 3D LVEF.
Nagata 2015 [25]	104	Asymptomatic severe AS with preserved LVEF (> 50%)	TomTec	TomTec	MACE or AVR (*n* = 33)	1) 2D GLS, 3D GLS, and 3D GRS were associated with outcomes. 2) AUC of 3D GLS was significantly larger than that of 2D GLS and 3D GRS. 3) 3D GLS was an only significant predictor after adjusting LV mass index and mean PG.
Sun 2016 [96]	66	Hemodialysis	Not performed	TomTec	MACE (*n* = 23)	3D GLS and 3D GRS were associated with MACE.
Casaa-Rojo 2016 [84]	45	Asymptomatic severe MR with Preserved LVEF (> 60%)	Not performed	Toshiba	MACE, LVEF< 60% or MV surgery (*n* = 15)	3D GLS, GAS, and GCS were associated with outcomes.
Shin 2016 [95]	96	Acute MI	Toshiba	Toshiba	MACE (*n* = 12)	3D GAS was associated with outcomes.
Howard-Quijano 2017 [47]	163	Cardiac surgery	GE	GE	MACE (*n* = 34)	All 3D global strains were associated with MACE.
Medvedofsky 2018 [97]	416	Diverse	Philips	TomTec	CV death (*n* = 114)	1) 2D/3D LVEF, 2D/3D GLS were significantly associated with outcomes. 2) 3D GLS was the strongest predictor for CV mortality.
Medvedofsky 2019 [98]	104	30–50% of 2D LVEF	Philips	TomTec	CV death (*n* = 32)	1) Not 2D LVEF/2D GLS and 3DLVEF but 3D GLS was associated with outcomes.

*AUC* area under the curve, *AS* aortic stenosis, *AVR* aortic valve replacement, *CD* cardiac death, *CV* cardiovascular, *LV* left ventricular, *LVEF* left ventricular ejection fraction, *MACE* major adverse cardiac event, *MI* myocardial infarction, *MR* mitral regurgitation, *MV* mitral valve, *PG* pressure gradient

Other abbreviations are the same in Table 1

### Novel techniques

Multimodality imaging allows fusion imaging [102–104]. Mor-Avi and colleagues performed fusion imaging using computed tomography coronary angiography (CTCA) and 3D longitudinal strain [103, 104]. Resting 3D regional longitudinal strain was color-coded to detect regional abnormalities in a 3D map. Stress CT perfusion was also color-coded to detect regional perfusion abnormalities. Fractional flow reserve (FFR) was also measured using CTCA. The presence of resting strain abnormalities had 71% sensitivity and 81% specificity for detecting > 50% stenosis in CTCA and stress-induced myocardial perfusion defects within specific coronary vascular beds. It had a sensitivity of 83% and a specificity of 81% for detecting stress-induced myocardial perfusion and FFR < 0.80. These results suggest that fusion imaging with 3D strain and CTCA provide valuable information to detect functionally significant coronary stenosis, even at rest. Additional large-scale studies are required to validate whether 3D regional longitudinal strain abnormalities at rest reflect solely reductions of coronary blood flow due to functionally significant coronary stenosis.

### Future directions

Like the 2D strain standardization taskforce [105–108], the American Society of Echocardiography/ EACVI and instrument partners should work together to identify causes of inter-vendor variability of 3D strain measurements with the objective of reducing it. Current 3D LV strain analysis is predominantly performed to evaluate global LV function. Segmental 3D strain abnormalities may represent specific pathologies. However, accurate 3D segmental tracking requires clearer 3D images with fine spatial resolution and high temporal resolution. Although one-beat acquisition of 3DE full-volume datasets using smaller 3DE transducers is now possible, spatial and temporal resolution are still not adequate to perform reliable segmental deformation analysis. This limitation also causes some underestimation of 3D global strain values, compared with corresponding 2D global strain values. Non-segmental (e.g., coronary artery territories) analysis of deformation is one solution to overcome current problems for segmental analysis [109]. Myocardial curvature analysis or LV shape analysis is another fruitful field of research, because it will provide useful information regarding disease severity and prognosis [99]. Since 3D strain simultaneously provides multidirectional strains, extraction of shear strain components which combine deformation in two different directions is also an interesting field for research [7, 110]. 3DE could be more useful than 2DE in complex and irregular heart chambers, like the right ventricle. 3D strain software aimed at other cardiac chambers is just starting and is now commercially available. It will soon be determined whether 3D RV global strains are more robust parameters than 3D RVEF to predict future outcomes, and whether 3D RV global strains detect subtle RV dysfunction in patients whose 3D RVEF is still normal. Finally, fully automated 3D strain analytical software will eliminate observer variability of 3D strain measurements, and will facilitate the use of 3D strain for routine adoption in the clinical arena.

## Conclusions

3D LV speckle tracking software simultaneously provides LV volumes, LVEF, and 3D global strains with multiple directions. It also provides a new 3D deformation parameter, such as area strain, which is an integral marker of longitudinal and circumferential function. However, 3DE data acquisition and subsequent strain analysis have not been widely adopted, and their evaluation is still limited to research applications. There are no consistent findings to suggest that 3D LV strain is superior to 2D LV strain in some clinical scenarios. In addition to facilitating further refinements of both 3DE image quality and 3D LV strain software, application of fully-automated 3D strain software may expand its adoption into routine echocardiography examinations.

## Supplementary information

**Additional file 1: Figure S1.** Forest plots of the mean difference in 2D GLS between patients with subclinical left ventricular dysfunction and control subjects (A), and corresponding 3D GLS values between the two groups (B) Each study shows first author’s last name, year of publication, and the reference number (parenthesis). CI, confidence interval; MD, mean difference; SD, standard deviation.

## Data Availability

The datasets used and analyzed during current study are available from the corresponding author on reasonable request.

## Abbreviations

2D: Two-dimensional
3D: Three-dimensional
2DE: Two-dimensional echocardiography
3DE: Three-dimensional echocardiography
CRT: Cardiac resynchronization therapy
CTCA: Computed tomography coronary angiography
EACVI: European Association of Cardiovascular Imaging
FFR: Fractional flow reserve
GAS: Global area strain
GCS: Global circumferential strain
GLS: Global longitudinal strain
GRS: Global radial strain
LLN: Lower limit normal
LGE: Late gadolinium enhancement
LV: Left ventricular
LVEF: Left ventricular ejection fraction
LVH: Left ventricular hypertrophy
ROI: Region of interest
