# Supplementary figures and images for: A review of current trends in three-dimensional analysis of left ventricular myocardial strain
Source: Cardiovasc Ultrasound. 2020 Jun 26;18:23. doi: 10.1186/s12947-020-00204-3 (PMC7320541; doi:10.1186/s12947-020-00204-3)

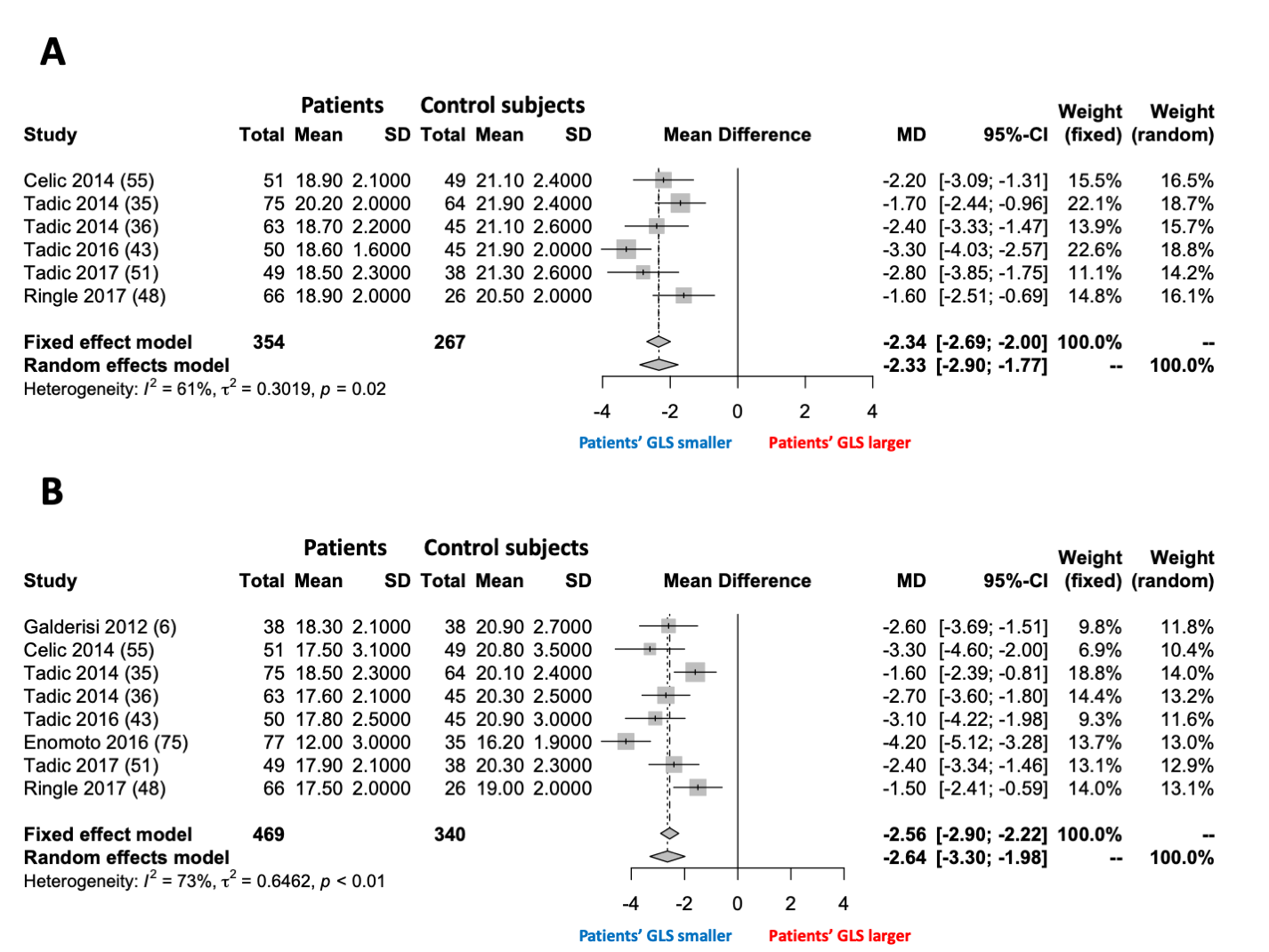

Supplement: Supplementary file 1 — Additional file 1: Figure S1. Forest plots of the mean difference in 2D GLS between patients with subclinical left ventricular dysfunction and control subjects (A), and corresponding 3D GLS values between the two groups (B) Each study shows first author’s last name, year of publication, and the reference number (parenthesis). CI, confidence interval; MD, mean difference; SD, standard deviation. [file 12947_2020_204_MOESM1_ESM.tiff]
